# Wanting to matter and learning to care: A neurodevelopmental window of opportunity for (Pro) social learning?

**DOI:** 10.1016/j.dcn.2024.101430

**Published:** 2024-08-14

**Authors:** Ronald E. Dahl, Emma Armstrong-Carter, Wouter van den Bos

**Affiliations:** a School of Public Health, Institute of Human Development, University of California, Berkeley, United States; b Tufts University, United States; c University of Amsterdam, the Netherlands

**Keywords:** Adolescent development, Prosocial development, Mattering, Pubertal maturation, Hypothalamus, Salience network

## Abstract

Wanting to matter—to feel socially recognized, appreciated, and capable of actions that benefit others—represents a fundamental motivation in human development. The motivational salience of mattering appears to increase in adolescence. Evidence suggests this is related to pubertal increases in the incentive salience for gaining social value and personal agency. This can provide a useful heuristic for understanding motivational proclivities (i.e. wanting to matter) that influence action-outcome learning as young adolescents are exploring and learning how to navigate increasingly complex social and relational environments. Adolescence also brings new capacities, motives, and opportunities for learning to care about and contribute to the benefit of others. Together, these create a window of opportunity: a sensitive period for learning to gain salient feelings of mattering through caring prosocial actions and valued societal contributions. Successfully discovering ways of mattering by doing things that matter to others may contribute to formative socio-emotional learning about self/other. Advances in understanding these social and relational learning processes and their neurodevelopmental underpinnings can inform strategies to improve developmental trajectories of social competence and wellbeing among adolescents growing up in a rapidly changing and increasingly techno-centric world.

## 1 Introduction: seeking a more integrative and actionable understanding

One set of considerations relevant to a special issue on *Developmental Cognitive Neuroscience in Society* focuses on how to advance a more integrative transdisciplinary understanding. That is, how to seek scientific progress that bridges between a discipline aimed at understanding the neural basis of cognitive and developmental processes and a set of related disciplines aimed more broadly at understanding learning and development—including the importance of social and societal contexts that can sometimes support, or at times, can undermine these processes. Ideally, aiming for integrative understanding that can lead to *actionable* scientific insights. In ways that can help to inform real-world efforts to improve developmental trajectories among the more than a billion adolescents growing up in complex and rapidly changing societies across the world.

This raises pragmatic questions. *How* to promote integrative transdisciplinary progress in ways that can inform real world challenges? Our aim here is not to advocate for a particular strategy or best path forward. Rather, it is to provide *an illustrative example of a promising approach*.

We describe a transdisciplinary approach that focuses on: a) a specific developmental transition at the onset of adolescence; b) dynamic multi-level changes related to biological maturation, social learning processes, and their interactions; c) how these, *together*, appear to create a window of opportunity for formative prosocial learning through ‘wanting to matter’ and ‘learning to care’; and d) considerations of how this approach can contribute to a more integrative understanding in ways that lead to *actionable* insights at the level of societal impact (Box 1).

Box 1 **Transdisciplinary Integration and Actionable Understanding.**

Over the past twenty years, the US National Institute of Health has advocated for the value of transdisciplinary approaches to advance progress to address complex health relevant challenges. Several reviews have sought to summarize the conceptual and definitional differences regarding *multidisciplinary*, *interdisciplinary* and *transdisciplinary* approaches to health research, services, education and policy (Choi and P, 2008, Choi and Pak, 2006, Choi and Pak, 2007, James et al., 2015, Sell et al., 2022). Areas of overlap are acknowledged with no single consensus definition; however, there is general agreement on key principles. Multidisciplinary approaches draw on knowledge from different disciplines but tend to recognize boundaries within each discipline. Interdisciplinary approaches tend to focus on the intersections between disciplines, with analyses and synthesis seeking a larger coherent whole. Transdisciplinary approaches seek to integrate evidence and insights from multiple approaches in ways *that transcend the traditional boundaries within disciplines.* By crossing disciplinary paradigms and integrating knowledge and perspectives from scientific and non-scientific sources alike (e.g., stakeholder groups, decision-makers, and participatory processes incorporating lived experience and youth perspectives) transdisciplinary approaches can contribute to more holistic understanding, leading to translational progress and/or *actionable* insights, applicable to complex real-world problems and challenges (Ciesielski et al., 2017, James et al., 2015).

Moreover, this appears to be a time of great opportunity for transdisciplinary integration relevant to the focus of this *Perspective*. Rapid advances in understanding processes of learning and development currently *span several different disciplines.* These include developmental cognitive neuroscience, developmental psychology, the science of learning, AI/machine learning and computational models of learning. These also include growing understanding of the quintessential roles of social, economic, cultural, familial, and other contextual factors that influence *how* learning and development occur—and the complex systems such as educational institutions, social media, and social networks that can scaffold and support or undermine learning and development.

## 2 The developmental transition from childhood into adolescence

When is a child no longer a child? Clearly, this identity-level transition cannot be adequately defined or understood from any single disciplinary perspective. The transition into becoming an adolescent involves a complex multifaceted set of developmental changes and learning processes.

From the perspective of biological maturation, this transition can be understood in terms of the physiological events that initiate a cascade of transformative growth and physical changes leading to sexual and reproductive maturation. From a psychological perspective, this transition may be understood in terms of acquiring new levels of knowledge, skills, and regulatory capacities for burgeoning social competence. From a sociological perspective, this transition may be understood in terms of social roles and relationships: gaining new freedoms, demonstrating more mature social behavior and responsibilities, and developing more mature relationships.

Yet, from an integrative, transdisciplinary perspective this transition might be understood as a dynamic period of multi-level *interacting* processes of learning and development. These include interactions spanning biological, psychological, and learning processes. This perspective is particularly relevant to understanding *social learning processes* (see Box 2) during this transition.

Box 2 **Social Learning Processes.**

Social learning encompasses a wide range of types of learning and is often defined differently across disciplines and fields. One common and inclusive definition entails all ‘learning that is facilitated by observation of, or interaction with, another individual (or its products)’ (Kendal et al., 2018). In psychology and behavioral ecology there has been a strong emphasis on skill learning through imitation behavior. However, a great deal of social learning involves advice taking and giving, teaching, and trial-and-error learning experiences while interacting with others.

Note that the goals of social learning can be social or non-social. For example, learning from social observations how to use a tool to crack nuts has non-social benefits (e.g. energy). Our primary focus here is on social goal-oriented learning from social experiences in interactions with others. In this case the affective response to evaluative feedback of the other is the crucial outcome and the focus of the learning behavior. In this context social learning can be based on simple trial and error, action-outcome learning, and the various ways of modeling these processes within the broad frame of reinforcement learning, (Olsson et al., 2020) including model-based processes that are used to infer the state of others minds (Jara-Ettinger, 2019). To accommodate social learning, basic reinforcement models are often extended with parameters to accommodate the informational (e.g. update belief about outcomes) and normative (e.g. positive experience of choosing the majority action) processes in social learning (Biele et al., 2009). Beliefs and social knowledge may change across development due to learning, but developmental changes in social and affective processes may be particularly important in understanding developmental changes in social learning in the transition into adolescence (Rodriguez Buritica et al., 2019). For example, consider the impact of a small pubertal increase in the salience of social evaluative feedback. This may begin to bias attentional processing (e.g. salience of social cues of acceptance, disapproval, being liked), but also, motivational salience (e.g. changes in the ways social experiences create motivationally significant events). Changes in motivational salience can gradually bias the selection of goals, behaviors, and emotional responses (social actions and reactions) to social feedback—in ways that can have cascading effects on action-outcome learning (Rodriguez Buritica et al., 2019, Wilbrecht and Davidow, 2024).

### 2.1 The transition into adolescence begins a new period of learning and development

The onset of puberty is associated with a broad range of social, affective, and motivational changes. These include increases in sensitivity to social feedback, sensation-seeking, novelty-seeking, risk-taking, and motivations to engage with peers. Together, these promote exploration and engagement with adolescents’ social learning contexts. This transition also presents new freedoms and opportunities as young adolescents are exploring and learning how to navigate the novel, uncertain, and increasingly complex social and relational contexts of *adolescence* (Ciranka & van den Bos, 2019; Ferguson et al., 2024). It is also a period of burgeoning social capacities and motivations for social and relational learning, (Allen et al., 2023; Crone et al., 2020; Suleiman et al., 2017). and for making larger contributions at a community or societal level (Fuligni, 2019).

Thus, this transition initiates a new period of *intertwined processes* of learning and development. Particularly for learning about self/other and self-other relationships and developing an adolescent social identity through action-outcome learning in day-to-day actions and experiences.

This framework helps to illustrate how a more integrative understanding of this transition can provide a promising approach to actionable insights*.* Because it: a) points to a developmental window of opportunity for formative social and relational learning; b) highlights the importance of a more integrative understanding of the changing *processes* of learning, motivation, and social experience feedback loops; and c) has implications regarding features of the social, societal, and technological contexts that can support and scaffold (*or* undermine) formative learning during this burgeoning period of social competence and identity development.

## 3 Wanting to matter: a promising focus for cross-level actionable insights

The motivation to matter—to feel socially recognized, appreciated, and capable of actions that benefit others—can be understood as an adaptive social learning heuristic for our species. This conceptualization may provide insights relevant to understanding fundamental aspects of social motivation in human development across the lifespan. Our primary goal in this *Perspective,* however, will focus on its relevance during this transition into adolescence.

Early investigations into mattering as a psychological construct in adolescence were carried out by Rosenberg and colleagues as they sought to expand the measurement of self-esteem to include aspects of self-concept shaped by a need to feel significant to others (Rosenberg, 1965, Rosenberg and McCullough, 1981).Work by Schlossberg and colleagues (Schlossberg, 1989) identified the importance of feeling appreciated as a core aspect of mattering. Over the ensuing decades research on the importance of mattering has expanded across the lifespan. There is evidence that feelings of mattering predict levels of wellbeing, self-esteem, and positive affect (Elliott et al., 2005, Flett, 2018, Jung and Heppner, 2017, Markus and Kitayama, 1991, Matera et al., 2020, Rosenberg and McCullough, 1981, Vélez et al., 2020) and *not* mattering is associated with depression and anxiety (Flett, 2018, Flett et al., 2016, Flett et al., 2022, Rosenberg and McCullough, 1981).

There are some ongoing issues regarding how best to conceptualize, define, and measure this construct (Flett et al., 2022, Reece et al., 2021) However, we highlight two cardinal features from the literature relevant to our heuristic model in this *Perspective.*

First, mattering is fundamentally *affective*—it has been assessed primarily in terms of a subjective sense of mattering to others (i.e. an internal appraisal about self, as being socially valued). This has important implications relevant to investigating its role in learning and motivation. *Wanting* to matter can be understood in terms of incentive salience (Berridge, 1996, Berridge, 2019) However, as we will discuss later, the motivationally salient ‘goal’ or outcome may be directly related to the personal subjective affective experience – i.e., a *feeling* of mattering.

Second, feeling socially valued is often related to (and can be assessed as) an appraisal of *action-dependent* outcomes: a subjective sense that one’s actions and efforts lead to outcomes that matter to others. This emphasis on the agentic dimension of mattering is described by Reece and colleagues (Reece et al., 2021) from evidence for a two-factor structure for mattering in adults: one related to feeling recognized/appreciated and the second to the perceived *impact* of one’s actions.

Taken together, these support a construct of mattering with two *interrelated* components: a) an affective appraisal in the domain of social value about *self*—a feeling that one matters to others; and b) an affective appraisal about the impact of one’s *actions*—a feeling that one’s actions and efforts matter to others). Our ‘wanting to matter’ heuristic in this paper focuses on how social learning processes can be motivated by *each* of these two *interrelated* components.

### 3.1 How a ‘wanting to matter’ motive shapes social learning and development

There is evidence that wanting, and actively seeking feedback relevant to *each* component of mattering begins to emerge quite early in development. Wanting attention and positive responses from caregivers is an axiomatic feature of early childhood (Hrdy, 2011) Infants demonstrate motivated behavior aimed at social goals—they *actively seek* attention and responses. Newborns can adjust the frequency of the sucking rate on a pacifier to obtain a socially salient outcome: a recording of their mother’s voice (Barrett & Morgan, 2018). This motivational salience for engaging attentive responses from a caretaker is reflected in the way one academic encapsulates a central insight of evidence-based parenting advice for 2–5-year-olds: *For our children, our attention is the ultimate positive consequence* (Nesi, 2024).

There is similar evidence for the early motivational salience in discerning if/how one’s *actions* impact others. The salience of response-contingent stimulation is evident from early infancy (Bigelow and Rochat, 2006, Rochat, 2001, Watson and Ramey, 1972). For infants and young children the salience of contingent social responses indicating a close association with one’s own actions underpins key aspects of moment-to-moment social learning processes. These processes have been demonstrated in event-related potentials to contingent mother-infant conversation (Lam-Cassettari et al., 2021) gaze-contingent eye-tracking to measure the reward value of social signals in toddlers (Vernetti et al., 2018) and the role of contingent social interactions in natural language learning (Masek et al., 2021).

Contingent responses are essential to action-outcome and motivational learning. In the domain of early social and relational learning, the salience of these action-based social feedback loops may be essential. Not only for social bond formation and natural language learning—but also to gain a sense of self, and social agency (i.e., an appraisal that one’s actions can have an impact on others).

While these motivational proclivities contribute to social learning through childhood, the transition into adolescence begins a new phase of social and relational learning. Social and motivational changes associated with puberty may play an instrumental role. As will be discussed in the next section, even a slight biologically based increase in the incentive salience for gaining social value and personal agency may begin to bias these learning systems. Also, puberty brings new motivational proclivities and capacities for social relationships and social bond formation.

## 4 Developmental changes in incentive salience and social learning

Changes in incentive salience can alter the value of a related outcome. In other words, changes in motivational salience can alter the types of learning experiences that lead to highly *desirable* outcomes. This is obvious in simple versions of incentivized action-outcome experiences. For example, a strong physiological thirst will increase incentive salience for drinking. This will change the relative ‘value’ of drinking a tepid glass of water—including its motivational value and the magnitude of reward/enjoyment it brings. Changes in incentive salience for social goals may similarly bias motivation, outcome value feedback of rewards, and related learning processes.

Consider for example, the effects on outcome value from a small (biologically based) maturational increase in an incentive for popularity or reputational gains among peers. Actions that lead to a reputational gain may be experienced as having greater value or priority (e.g. compared to a monetary gain). Gaining reputation and gaining money are strong reinforcers for young adolescents. A study of 10–13 year old girls performing an Auction Task found that pubertal maturation was associated with patterns of over-bidding behavior that prioritized reputational over monetary gains (Cardoos et al., 2017). This finding is consistent with other evidence of a developmental shift in early adolescence to prioritize positive peer evaluations over other personal and academic goals (LaFontana & Cillessen, 2010).

As will be discussed in the next section, we believe that emerging evidence is consistent with a hypothesis that pubertal maturation is associated with an increase in the incentive salience for earning social value—in ways that increase the salience of outcomes (or cues predicting outcomes) of gaining or losing social value. Even small shifts in motivational salience, can gradually accrue large effects across hundreds of moment-to-moment and day-to-day social learning experiences.

### 4.1 Socio-motivational changes in the transition into adolescence appear to contribute to increased motivational salience for gaining social value

There has been considerable progress in understanding social and affective changes during adolescent development. A central theme focuses on the attentional and motivational salience of peers—including an increased prioritization of social cues, social information, social goals, peer contexts, and sensitivity to social hierarchies, status, and popularity. (Blakemore, 2008, Blakemore et al., 2010, Blakemore and Mills, 2014; Capella et al., 2023, Crone and Dahl, 2012, Crone and Fuligni, 2020, Dahl et al., 2018; Dai et al., 2023, Foulkes et al., 2018, LaFontana and Cillessen, 2010, Molleman et al., 2022, Nelson et al., 2016, Nelson et al., 2005; Perino et al., 2016, Pfeifer and Berkman, 2018, Somerville, 2013, Telzer et al., 2021).

A second closely related theme has focused on emotional sensitivity to social evaluation—particularly in response to social rejection feedback (Achterberg et al., 2017, Gunther Moor et al., 2010, Masten et al., 2013, Silk et al., 2014, Silk et al., 2012). Some studies ((Silk et al., 2014) have demonstrated the specific association between pubertal maturation and this increased sensitivity to social rejection. Several recent investigations (Dalgleish et al., 2017; Davis et al., 2023; Dobbelaar et al., 2023; Van de Groep et al., 2021) have also emphasized a broader *social salience* perspective on this sensitivity—demonstrating that both social exclusion and social inclusion show similar activation of dACC and anterior insula (the primary nodes in the Salience Network).

This broader, social salience perspective highlights the enhanced sensitivities to social learning cues as potentially contributing (in *adaptive* ways) as adolescents are learning how to navigate moment-to-moment social interactions that require rapid subjective evaluation and responses in real time. This is also consistent with emerging understanding of the development of social competence as the ability to fulfill personal goals in social interactions while also maintaining positive relationships with others over time and across situations. That is, by learning to balance goals related to one’s own wants and needs with the wants, needs, and goals of others (Crone et al., 2020, Crone et al., June 2024, Van Der Meulen et al., 2023).

There is emerging evidence to link this increased sensitivity to social evaluative feedback (and its association with pubertal maturation) to changes in salience processing. For example, a recent study used a task where adolescents believed they were being intermittently evaluated by peers and found puberty-specific changes in salience network connectivity (Pelletier-Baldelli et al., 2024). The authors interpreted these findings as consistent with a model of an evaluation-sensitive period of social learning.

Taken together, these findings provide support for a central premise in our heuristic model: the onset of puberty is associated with a developmental increase in the incentive salience for gaining positive social feedback—about one’s value. This motive—wanting to *feel* that one has value to others—becomes more salient in adolescence. Social learning experiences that activate these desired feelings of *mattering to others* are more likely to be experienced as motivationally salient, reinforcing the specific actions and efforts that preceded this desired outcome.

The degree to which these actions *also* create positive feedback for personal agency may further amplify the reward value. As described, the onset of adolescence appears to increase the incentive salience for each component: wanting to feel valued *and* to feel that one’s actions matter to others.

Thus, *agentic prosocial behaviors* can create positive feedback relevant to both amplified incentive systems. One can discover ways to gain feelings of mattering *by doing things that matter to and for others*. This can motivate efforts to seek these desired feelings through other versions of agentic prosocial behavior. These rewarding social actions can create strong and motivationally salient, positive feedback loops. In ways that can lead to feeling appreciated *and* building an internal appraisal that one’s actions can make a small or large impact that can benefit others.

## 5 The potentially pivotal role of prosocial (identity) learning as a way of mattering

One aspect of this ‘wanting to matter’ motive warrants further scrutiny. The definition—*wanting to feel socially recognized, appreciated, and capable of actions that benefit others*—focuses on *prosocial* actions. Yet there also are many ways that selfish and *antisocial* behavior can provide agentic social feedback on a day-to-day basis. For example, when young people receive attention and responses for bullying or excluding a peer. Over time, these can contribute to a self-appraisal that one’s actions are capable of impact (Hensums et al., 2023).

Humans display a remarkable capacity for two, quite opposite, socio-motivational extremes. At times, a truly inspiring capacity for caring, generosity, and cooperation. But also, an equal capacity for selfish Machiavellian intelligence, hoarding of goods, striving for status and power, incessant wars, subjugation of the weak, and growing threats of destroying each other, and our planet. On a smaller scale, and from a developmental perspective, antisocial daily acts can take the forms of relational aggression, harmful gossip, or strategically undermining other’s efforts.

There is compelling evidence that our species possesses a truly unique *capacity* for prosocial motivations and proclivities (Hrdy, 2011, Silk and House, 2011, Tomasello and Vaish, 2013). Moreover, key underlying features of prosociality such as shared intentionality, intrinsic motivations to help others, and proclivities for intersubjective experience, emerge very early in human development—some beginning in infancy (Higgins, 2016, Tomasello, 2019, Tomasello and Carpenter, 2007, Wolf and Tomasello, 2023) and others by the second year of life (Brownell et al., 2013, Paz et al., 2023).

These early prosocial motives appear to have biological underpinnings, (Warneken, 2016) are further shaped by early social learning experiences (Blake et al., 2015, Grueneisen and Warneken, 2022) and continue to develop through middle childhood (Grueneisen and Warneken, 2022, Song et al., 2022).There also appears to be a developmental shift from *intrinsic* concern for helping at age two, to more strategic (i.e. reputational) motivations for helping by age five (Hepach et al., 2023). This and related research point to potential windows of vulnerability/opportunity for *modifying* prosocial developmental trajectories, such as the transition to early schooling around age five (Hepach et al., 2023) and in adolescence (Fuligni, 2019).

Research has documented substantial variability in children’s prosocial motivations, and how these develop across infancy, childhood, and adolescence (Carlo, 2022, Carlo and Padilla-Walker, 2020, Eisenberg et al., 2015, Eisenberg et al., 2016; Fuligni, 2019, Grueneisen and Warneken, 2022, Rogoff, 2014, Tomasello, 2018, Warneken and Tomasello, 2009). Prosociality also varies considerably within and across cultures (Carlo and Padilla-Walker, 2020, Eisenberg et al., 2015, Rogoff, 2014) highlights the role of “children’s initiative in contributing responsibly to ongoing activities of their families and communities” especially in collectivist societies (Rogoff, 2014). This points to the importance of social and relational contexts that can scaffold and encourage culturally-meaningful aspects of social learning as integral to prosocial development, in ways that are both structured but also allow for individual agency.

**Fig. 1:**
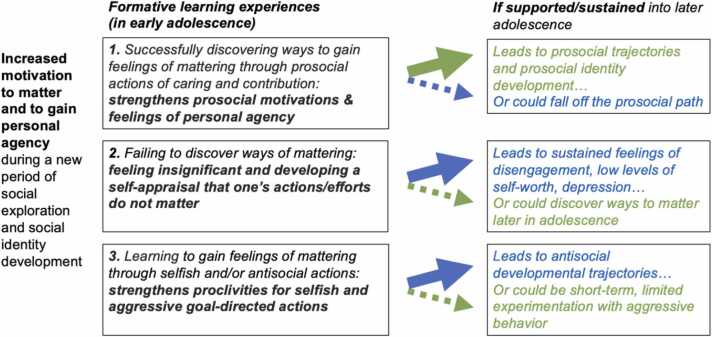
Transition from childhood into adolescence as a pivotal time of social-affective learning and identity development: Three examples of how ‘wanting to matter’ may influence trajectories:.

This evidence returns us to the primary focus here: how the *transition into adolescence* creates fundamentally new—and potentially pivotal—opportunities for prosocial learning (see Fig. 1). In broad terms, this includes the emergence of new capacities and proclivities for: a) caring about *and* connecting emotionally with others; and b) contributing in valued ways at a community or societal level.

## 6 Learning to care and connect emotionally: developing intrinsically motivated prosocial behavior

What underpins *intrinsic* prosocial motivation? *Wanting* to give and share generously? Some highly prosocial individuals seem to possess strong natural proclivities to act in ways that benefit others—and to find these experiences intrinsically rewarding. Among groups of people who care deeply about each other (e.g. family, close friends, or others with shared emotional connections) it is not uncommon to feel intrinsic motivation for giving generously. *Feelings* of caring and emotional connection are strongly associated with intrinsic motivations for prosocial behavior.

One might hypothesize a deeper causal relationship. That is, as an adolescent develops strong feelings of caring about a friend, these could contribute to intrinsic motivations to act in helpful supportive ways toward this friend—*in part, because such actions tend to feel rewarding.*

Consider for example, the early stages of the social learning processes that underpin the development of a *new* close and caring relationship, such as two adolescents exploring a mutual attraction. Even an innocent infatuation or ‘crush’ can activate very strong feelings of caring. Leading to affective appraisals of *valuing* each other and wanting to give and share generously.

As we start to care strongly about someone, this person becomes more salient. We are more likely to notice, respond to, empathize with, and prioritize this person. Feelings of caring can motivate concern about this person’s feelings—their wellbeing and happiness. We are more likely to offer kindness, generosity, support, or caregiving behavior *because* we have developed strong feelings of caring. Learning to care—through moment-to-moment and day-to-day experiences—appears to involve affective processes that can include feelings of caring that promote prosocial motivations.

Moreover, this example of developing an emotional connection through mutual or reciprocal caring helps to illustrate how wanting to matter and learning to care *can work together*—*synergistically.* Feeling that we matter to someone we care about can be deeply rewarding. In ways that can motivate more caring and giving. Actions that express these feelings (e.g. caring and prioritizing) tend to create authentic feelings of mattering—and when these feelings are reciprocated, can be powerfully rewarding (and powerfully motivating) emotional experiences.

This is not limited to the social learning processes that underpin the development of romantic relationships. Humans have a remarkable capacity for developing emotional connections and deeply caring relationships—in myriad ways, throughout the lifespan.

In our species, however, evolution appears to have favored a life history pattern that tends to prioritize this motivational salience (*wanting* to form emotional connections) during two key developmental windows: 1) as infants, actively seeking connection and attachment to caretakers; 2) as adolescents, with newly emerging capacities and motivations for learning to care deeply and to form strong emotional connections (extending beyond family).

This framework brings us back to considering the importance of advancing a more integrative understanding of the developmental social neuroscience and social learning processes that begin to change during this transition from childhood into adolescence. Including the importance of developmental changes in incentive salience and social value feedback loops. As with the earlier example of adolescent capacities for infatuation, it can be striking to consider how quickly these social *relational* learning experiences can rapidly *transform motivational priorities in adolescence*.

Not only can young adolescents develop intense infatuation with individuals they have never met (e.g. a celebrity) or barely know. But also, when reciprocated, strong mutual feelings of caring and emotional connection can accelerate rapidly. In ways that can create intense motivations.

This reciprocity illustrates the capacity for *amplified positive feedback loops* in these emotionally charged adolescent relational learning experiences. In contrast to the formation of parent-infant bonds which have quite asymmetric processes of caring and responsiveness, the social learning processes in early-stage romantic relationships tend to have a more co-created, mutually agentic, interpersonal dynamic. This can create *equally reciprocated* actions of affection, giving, caring, and activating powerful feelings of emotional connection and mattering to each other. This also includes new (sexual) capacities for conveying and activating strong feelings, along with a wide array of complex planful, and goal-directed social behavior to convey this prioritization.

This can create a remarkable capacity for high-intensity *transformational* changes in motivation. As is well illustrated by the age-old story of two teenagers, smitten with each other, who start to believe the entire universe has destined for them to be together and are soon willing to abandon their families and everything they ever valued. They would prefer to die rather than live apart—even though they just met two weeks earlier and barely know each other. Yet, rather than an absurd comedy, *Romeo and Juliet* is perhaps the most enduring romantic *tragedy* in literature.

The implications here go well beyond a developmental increase in ‘wanting to be popular’ or motivations for gaining social reputation. This capacity for social learning processes to create rapid transformational changes in personal motivational priorities suggests a developmental window of opportunity for *learning to care* in fundamentally new ways. Because caring about and prioritizing another person can be difficult, effortful, and risky—but, if done successfully in a relational giving-and-receiving way, can satisfy needs for caring *and* mattering.

### 6.1 Learning to care, caregiving relationships, and skills for developing social competence

Crucially, this shift in the motivational salience of developing deep feelings of caring about someone can make it easier to prioritize caring *actions*—caregiving behaviors. *Actively* caring can be effortful and burdensome. That is, attending to the needs, wants, feelings, and goals of someone else, can delay, or displace the ability to serve one’s own needs, wants, and goals.

As described earlier, emerging understanding of the development of social competence in late childhood and the transition into adolescence has provided support for potentially advantageous balance of some aggressive and prosocial strategies to achieve social goals (Hawley, 2003, Hawley, 2014, Hawley et al., 2007) in ways that can lead to socially successful trajectories (Dobbelaar et al., 2023; Hartl et al., 2020).

For adolescents, learning how to balance these often-competing priorities may be an important component of developing social competence. Consider the bidirectional aspects of developing feelings of caring *and* caregiving behavior. Caretaking responsibilities for a person to whom one *lacks* any emotional connection can feel burdensome. Even within a supportive family, excessive caretaking responsibilities can interfere with school, homework, and social learning with peers (Armstrong-Carter et al., 2021, Caregiving Youth Research Collaborative, 2023, East, 2010, Hooper et al., 2014, Siskowski, 2006). Yet, early caregiving experiences can also create valuable opportunities—learning to care for and about others in ways that contribute to feelings of connection and mattering.

Caregiving can be or can become intrinsically rewarding—particularly when facilitated by motivationally salient feelings of caring. When we have developed strong feelings of caring about someone, we may begin to care about their needs, wants and goals as feeling personally salient. Serving those needs may still require effortful actions, but also, in ways that can *increase the rewarding nature of serving those needs*. This can motivate more prosocial behaviors.

These social learning experiences, including caregiving and family contribution, are important dimensions of social development throughout childhood (Armstrong-Carter et al., 2019, East, 2010, Fuligni, 2019). Yet, the transition into adolescence brings new capacities for learning to care (Chase-Lansdale et al., 1995, Lockwood et al., 2024) and wanting to matter—along with new motivations for emotional connection and sharing intimate information (Nowell et al., 2023). Moreover, caregiving success in early adolescence predicts social and relational functioning as adults (Allen et al., 2023). The development of caregiving skills during childhood and adolescence might serve as a crucial segue to the childrearing or eldercare responsibilities characteristic of adulthood.

Also, in ways that parallel this capacity for rapid relational learning, the transition into adolescence also appears to create opportunities for learning to care deeply in other ways—through valued contributions. A capacity to develop heartfelt, intrinsically rewarding goals. Learning to care deeply about something larger than self—in ways that may promote intrinsically rewarding *prosocial* motivations. Wanting to contribute to communities, societies, or meaningful causes.

## 7 Learning to matter through contributions at a community or societal level

The transition into adolescence also brings new capacities and motivations for contributing in small and large ways on multiple levels—not only helping and supporting peers and family but also actions that benefit others at a community or societal level (Crone and Fuligni, 2020, Fuligni, 2019). These contribution activities may or may not have the same (directly) *relational* quality as caring about and for another person, however, these prosocial actions can create similar positive feedback loops. That is, an adolescent can discover ways of gaining these desirable feelings of mattering through socially valued contributions—in whatever manner of giving that begins to feel personally meaningful to them.

For many young adolescents who are transitioning to peer contexts outside of the family, this creates a wealth of opportunities for discovering ways that prosocial actions can lead to rewarding feelings of mattering. For example, young adolescents may find a deeply rewarding experience by supporting a friend in distress, or by contributing to a personally meaningful cause such as climate change or social justice (Armstrong-Carter and Telzer, 2021, Fuligni, 2019). These can occur as individual contributions or through collective action as part of a group. Yet, as others have noted, adolescents growing up amidst social, economic, and racial inequities tend to have fewer opportunities for meaningful contribution (Crone and Fuligni, 2020, Fuligni, 2019, Gaby, 2017).

These forms of prosocial learning experiences may shape individual development in formative ways during this pivotal transition into adolescence. As young adolescents discover ways of caring and contributing that are personally and culturally meaningful to them, these experiences can feel deeply rewarding in three synergistic ways: 1) activating salient feelings of mattering; 2) providing positive feedback that one’s actions matter; and 3) through positive *prosocial* reputational effects—feeling appreciated for caring/contributing to something larger than oneself.

Amplified versions of rewarding (and motivating) feedback for prosocial actions may create opportunities for pivoting *away* from alternative trajectories. For example, preventing an impairing cascade of anxious and sad feelings from a sense of not mattering. Or diverting away from antisocial pathways for gaining feelings of agency and impact. [As illustrated in Fig. 1]

Deeply rewarding *prosocial* versions of mattering in early adolescence might also create some healthy alternatives for adolescents growing up in hyper-competitive social contexts. Social contexts that place extreme value on elite achievement can create adolescent perceptions of a very narrow path for gaining social value—in ways that can create fierce status competition and extreme stress related to pressures to achieve in academics, sports, for on-line fame, or financially. This also raises important questions about some generational shifts in societal values and escalating achievement expectations (Curran and Hill, 2022, Luthar et al., 2020) that might be inadvertently undermining prosocial learning.

These examples of potentially modifiable developmental trajectories of prosociality help to illustrate the potential value of advancing a more integrative scientific understanding of these and related social learning processes—in ways that can be leveraged for positive impact. This will require advances in research at the level of developing neural systems and developmental changes in feedback loops, but also in terms of actionable insights and testable hypotheses relevant to societal level impact. We will briefly sketch a few illustrative examples at each level.

## 8 Research implications focusing on developing neural systems, developing social learning systems and cross-level feedback loops during the transition into adolescence

First, we highlight the need for a deeper mechanistic understanding of maturational changes that appear to bias social learning processes in adolescence. There is currently ongoing progress in several areas (across disciplines). Our goal here is to suggest some promising approaches to help inform a more integrative and actionable understanding.

For example, incorporating a stronger focus on the hypothalamus as an integrative neural hub that is directly involved in the onset of puberty may provide new insights into how maturational changes can bias behavioral and learning priorities. These hypothalamic influences likely occur through interactions with other neural systems and networks. Progress will require a transdisciplinary approach—in ways that consider interactions across hypothalamic regulatory systems, including energy balance, sleep and circadian, and changes in social motivations. Given recent basic science advances, (Maroso & Stern, 2023) this could create exciting collaborative research opportunities from transdisciplinary teams focusing on a more integrative and insightful understanding of pubertal hypothalamic changes influences focusing on *social* learning in human development.

A second (related) area of opportunity focuses on the value of a stronger emphasis on the *affective* dimensions underpinning social learning processes and social feedback systems. Understanding how these undergo change during the transition into adolescence, including the role of neural signals that we experience as behaviorally and motivationally relevant feelings, could provide new insights into shifting behavioral priorities and motivational salience in adolescence. This focus on affective signals and neural systems is relevant to the focus on hypothalamic inputs because behavioral priorities related to homeostatic goals often operate through affective signals.

Consider, for example, the motivationally salient feeling of thirst. A feeling of thirst is not a sensation of having a dry throat—rather, it is the product of an elegant set of computational processes monitoring information from several physiological sources, particularly the delicate balance of sodium and water molecules in our blood. This complex flow of information is being summarized, constantly, in real time, to produce a moment-to-moment output signal. A feeling. Recognizable and motivationally salient: *Thirst.*

This provides a vivid example of a hypothalamic regulatory output that can activate and prioritize a specific signal of *wanting.* Crucially, we experience this *wanting* in two ways: as a recognizable feeling of thirst and as a specific motivation or action tendency: *seeking water*. A subtle feeling of thirst may be barely perceptible—simply an action selection to sip from a fountain without any conscious thought of having been thirsty. A larger physiological imbalance creates a stronger signal—a level of thirst that can become hard to ignore. As physiologic imbalances approach a danger point these signals intensify into a feeling that can compel our every thought, planning and action to focus, unwaveringly, on a single goal: water.

Clearly, multiple competing motivational priorities need to be traded-off dynamically, on a moment-to-moment basis, and balanced over longer time scales. We need to continuously weigh hunger and thirst, needs for sleep, and temperature regulation against competing social needs, such as social connection and achievement goals, according to our current *state* and *opportunities in the environment.* The hypothalamus plays a key role in trading-off these different motives (Petzold et al., 2023). One way of describing the role of the hypothalamus in prioritizing different behaviors is by modulating the gain on certain signals, thus altering the field of affordances (Jabarin et al., 2023).

This example helps to frame how a subtle hypothalamic influence on motivation near the onset of pubertal maturation might operate through increased gain in signals related to social evaluative feedback. The hypothalamus is an integrative regulatory hub, which occupies a central role in puberty. A slight amplification in the gain on social signals can increase the drive towards social connection. Moreover, other interrelated homeostatic regulatory processes also have hypothalamic influences—creating opportunities for adaptive shifts in behavioral priorities to serve biological as well as social priorities (See Box 3).

Box 3 **Understanding hypothalamic and pubertal influences on social motivation and learning.**

Puberty involves a set of interrelated hypothalamic and neuroendocrine systems (Naulé et al., 2021). These include three set of component processes with somewhat different developmental timing, different hormonal mediators, and different physiological and maturational consequences (e.g. gonadal maturation, acceleration of physical growth, and secondary sex changes related to adrenal androgens). In addition to interactions between/among these component systems, there is a growing understanding of *a broader set of interrelated* changes in hypothalamic regulatory systems that are involved in motivation, learning, and behavior (please see *Science special issue* (Fong et al., 2023, Maroso and Stern, 2023) and (Naulé et al., 2021) for helpful figures and much more in depth discussion]

To illustrate some of the pragmatic and theoretical challenges these raise, let us focus on one major component: the Hypothalamic-Pituitary-Gonadal axis (Fong et al., 2023, Maroso and Stern, 2023, Naulé et al., 2021). One critical *initiating* event involves pulsatile release of kisspeptin (from Kiss-1 neurons in the hypothalamus) that acts on Gonadotrophin Releasing Hormone (GnRH) neurons to create a pulsatile pattern of release of GnRH. Critically, the pulse *frequency* of hormone discharge is the key activating signal to the pituitary. The pituitary responds with pulsatile release of Luteinizing Hormone (LH) and Follicle Stimulating Hormone (FSH) into the bloodstream, which gradually activate maturational changes in the gonads (testes and ovaries). This process (along with the next-level transformative changes caused by gonadal hormones circulating through the body) proceeds over a span of years.

How might these details help to inform a research question such as: *how does the onset of puberty influence sleep and circadian regulation?* This question is highly relevant to real world issues impacting adolescent sleep health, such as school start times, (Minges and Redeker, 2016, Morgenthaler et al., 2016) and clinical research into the role of sleep problems in adolescent vulnerabilities for developing anxiety and depression (McMakin and Alfano, 2015, Sadeh et al., 2009). A typical approach might be to examine how gonadal hormone levels influence neural systems involved in sleep-wake and circadian systems (Fong et al., 2023, Maroso and Stern, 2023). However, these same sleep and circadian systems are *also* directly involved in the *upstream* hypothalamic events that *initiated* puberty (Lucien et al., 2021). For example, the key signal (release of GnRH in a pulsing frequency q 60–90 min) typically begins while children are asleep. Moreover, these hypothalamic sleep and circadian systems are interrelated with other neuroendocrine processes of puberty (Fong et al., 2023, Maroso and Stern, 2023). In children the release of pituitary growth hormone occurs primarily during deep slow-wave sleep, and a key aspect of the pubertal growth spurt is caused by gradual increases in the amplitude and frequency of growth hormone pulses. Activity in the Hypothalamic Pituitary Adrenal axis (stress/cortisol) is closely linked to systems involved in REM sleep and circadian regulation (O’Byrne et al., 2021) and the physiologic architecture of sleep (alternating between periods of REM and non-REM) also follows 60–90 min cycles (the same as the signaling frequency of GnRH).

The primary implications focus on the multiple *bidirectional* interactions between sleep-wake regulation and the onset of puberty—including some that begin well *before* any measurable downstream effects of gonadal hormones. This general principle has important conceptual *and* methodological implications. For example, if one wants to test a hypothesis that focuses on the initiating events of puberty one could prioritize measures with closer associations with those events (e.g. nocturnal release of LH from the pituitary, which can be detected in the urine and may be one of the first measurable indications that central puberty has begun; Demir et al., 2023).

There are also bidirectional interactions with *metabolic regulation and energy balance* (Anderson et al., 2024). High levels of physical activity and low energy stores (e.g. very lean body mass) can directly delay the onset of puberty; *and* once puberty has begun pubertal growth spurt impacts metabolic systems of growth and energy storage. There are also bidirectional effects involving *the Hypothalamic-Pituitary-Adrenal axis*: social stress can influence pubertal timing in complex ways; *and* pubertal maturation has direct effects on cortisol regulation and social stress responses.

Taken together these examples support an integrative (transdisciplinary) perspective on puberty—framed broadly as a *complex multifaceted transition*. This also highlights the value of conceptualizing this transition (into adolescence) in terms of changes in *multiple interacting feedback systems* involving several different (developing) regulatory systems.

This conceptualizing helps to frame the primary focus for this paper: the need for a deeper and more integrative understanding of the interactions between pubertal maturation and developing neural systems involved in social motivation and learning.

In addition to the focus on hypothalamic influences, this emphasis on feeling-based neural signals that can shift behavioral priorities raises questions focusing on pubertal changes in salience processing. More specifically a stronger focus on the anterior insula (as a key hub within the broader midcingulo-insular *salience network*) may play a crucial role in shifting priorities during windows of learning and development. As described in Box 4 there is growing evidence of the importance of this network not only in ‘feeling-based’ learning processes, and in individual differences in salience and ‘caring’, but also in relation to prioritization of behavior through executive control in concert with the hypothalamus. Feelings may play an important role in moment-to-moment value based action selection in complex social situations (Durlach et al., 2002, Hall et al., 2000, Jung and Heppner, 2017).

Box 4 **A neural system for “anything a human might care about”:.**

**(Feelings, the anterior insula, Salience Network, and** ***learning to care)***

Salience processing plays a critical role in prioritizing the flow of information in the human brain. The Salience Network is involved in continuously monitoring multiple channels of physiological and sensory information. This is an essential part of *homeostasis*—a set of regulatory processes that maintain the delicate balances in physiological states essential to support life. These are monitored, dynamically, in real time. Moment-to-moment. As significant imbalances are detected, this salience system responds with output signals that we experience as feelings. A salient feeling of severe thirst, for example, communicates a high priority behaviorally relevant signal—and provides motivation for adjusting our behavior accordingly.

However, in addition to this role calling attention to high priority information relevant to our *internal* bodily states, this Salience Network is also critical in evaluating—and calling attention to—high priority information from the *external* world around us. Including salient physical, emotional—and in many situations—social information.

Historically, in the first scientific publication to identify this set of intrinsically connected pathways in the brain and name it (Seeley et al., 2007), Seeley and colleagues identified two general networks—an executive control network and another they labeled ‘the salience network’. Over the ensuing decade, a multitude of neuroimaging studies kept implicating an ever-broadening range of ways to activate this salience network: thirst, hunger, pain, bladder distention, embarrassment, uncertainty, amusement, compassion, tenderness, humor, emotionally moving music, the faces of loved ones, social exclusion, trust, empathy, sculptural beauty, a ‘state of union with God’, a hallucinogenic state (induced by ayahuasca) and tasting wine (in wine-lovers).

Twelve years later, reflecting back on that work, Seeley (2019) put it this way: “Many authors in their respective fields had begun to write about the ACC and AIC as key hubs of a [fill-in-the-blank] network, where the blank could be filled by **almost anything a human might care about**.”

The *salience network* does, indeed, seem to be involved in virtually anything a human might care about. Or, to reframe this slightly—from a developmental perspective—virtually anything a particular person may have *learned* to care about.

This conceptualization of salience as a fast prioritization system for noticing and responding to personally significant cues, raises compelling questions about the learning processes that shape personal salience. There is a growing literature focusing on these questions from a learning and development perspective (Menon and Uddin, 2010, Molnar-Szakacs and Uddin, 2022). Interestingly, some researchers have been emphasizing the role of these feeling-based prioritization processing in the anterior insula as highly relevant not only for attentional processing but also for prioritization for action selection and executive control (Molnar-Szakacs & Uddin, 2022).

Learning how to manage and respond to amplified ‘feeling’ signals of salient social feedback may also play a role in developing social competence. This could include learning to calibrate the intensity of affective signals in response to highly salient social and emotional experiences, as well as the development of motivationally salient feelings when learning to care deeply about others.

Another related set of research implications focuses on new insights into formative learning. For example, perhaps surprisingly, even the simple prioritization of water-seeking behavior in response to thirst is not purely instinctual. Animal studies demonstrate that this motivation requires at least some learning in the form of searching for and finding water *under a condition of thirst* (Berridge, 1996, Hall et al., 2000).

Thus, the outcome value is largely determined by how it impacts the *quenching* of thirst. Importantly, this can contribute to the development of enduring individual differences in preference (e.g. for a certain kind of drink) through highly salient early learning experiences. For example if young animals quench their thirst with flavored sugary water made available during initial bouts of thirst, they will continue to prefer this even if other more nourishing sources become available at later stages (Durlach et al., 2002).These initial motivationally salient learning episodes can thus serve as ‘formative’ experiences by shaping strong preferences for *how* one meets a category of needs (Durlach et al., 2002).

This simple example raises questions about potentially formative learning experiences as *new* social motivational priorities are emerging. In contrast with the water-seeking incentive from thirst, a motivational feeling of wanting to matter or gain personal agency could have a diverse range of potential sources of fulfillment. For example, formative experiences with antisocial day-to-day experiences of mattering. Or perhaps an absence of opportunities to meet these social needs could contribute, in formative ways, to a (helpless) affective appraisal that one’s action will not matter.

Finally, one set of compelling implications focuses on the need for more integrative models of reinforcement learning—*in ways that incorporate social and affective processes in new ways*. There is increasing recognition of the importance of not only considering objective value of expected outcomes in learning models, but also a broader recognition of the inherently *affective* nature of comparing relative value in moment-to-moment prioritization of actions (Juechems and Summerfield, 2019, Keramati and Gutkin, 2014, Vollberg and Sander, 2024). This emphasis on a specific affective state, both as goals and outcomes, has important implications at the level of reinforcement learning models—and how these change with development.

Most current learning models assume learners have static goals. Although it is well established that across development, model parameters such as learning rates may change (Nussenbaum & Hartley, 2019) it is usually assumed that the value of the outcome, whether it is money or points, is constant across tasks and developmental phases (i.e. more is better). However, the current perspective highlights how the subjective value of outcomes is dependent on current needs and goals, as well as earlier formative learning experiences. When integrating affective states in reinforcement learning models, the goals of learning are not to acquire as much of a good as possible, but rather, learning to acquire the right amount at the right time integrating over different timescales. This has potentially powerful implications for understanding social and relational learning—particularly in a formative window of adolescent development.

Notably, a goal for a learning algorithm can also be an affective state, like feeling satiated or rested (Bach and Dayan, 2017, Emanuel and Eldar, 2023). Also, recent learning models have started to integrate concepts of mood state into reinforcement learning models, suggesting that mood represents the momentum towards reaching goal states (Eldar et al., 2016). That is, when consistently receiving positive prediction errors, indicating a situation is better than expected, mood will rise (and vice versa).

Given that affective experience, including mood state, is hypothesized to bias the interpretation of outcomes consistent with the current moods, these affective states will influence the strength of feedback loops. Consequently, a positive mood will increase the motivation to engage with the environment, whereas low moods will lead to disengagement. When interacting with another person, a series of positive reciprocal actions can thus lead to a quick rise in mood and further motivation to engage, but a strong affective response to a single negative statement may lead to disengagement. A more integrative understanding of affective influences on social learning may provide insights into the rapid increase in engagement (or disengagement) that can be typical for adolescent social interactions. Social learning (about self, others, relationships, and societal values) is likely to involve rather complex computations about value—and in ways that may be changing with pubertal development.

## 9 Research implications focusing on interventions, implementation, and policies

First, we return to a core principle described in the introduction: the value of transdisciplinary teams with expertise spanning requisite domains to advance integrative understanding that can lead to actionable insights. But here, the goals aim for insights that are *testable*—at the level of interventions and policy. An example can be helpful to illustrate some principles.

Potentially actionable insights into adolescent sensitivities to social evaluative influences (combined with a review of ineffective school-based behavioral health interventions in adolescents) led Yeager, Dahl, and Dweck (Yeager et al., 2018) to propose a model predicting that interventions with values-aligned messaging could more successfully influence adolescent behavior. A pre-registered, longitudinal, randomized, controlled field experiment was designed to test this approach with an intervention to promote healthy eating (Bryan et al., 2019). The value-aligned intervention focused on shifting away from traditional health advice messaging (e.g. communicating what adolescents ‘should’ do to become healthy eaters, which can *feel* a bit condescending to adolescents). Instead, the messaging strategy focused on honoring adolescents’ desire for admiration and respect (e.g. *healthy eaters are strong-minded independent thinkers who stand up to the junk food industry*). This succeeded in substantially improving daily dietary choices in the school cafeteria (Bryan et al., 2019).

Galla and colleagues (2021) used a similar approach in two preregistered experiments with 2733 U.S. students ages 13–19 years to evaluate an intervention using a values-alignment message to improve adolescents’ motivation to control social media use. Results offer support for leveraging adolescents’ drives for autonomy and social justice to motivate self-regulation of social media use, particularly for girls. These findings fit into an emerging developmental understanding of social media use and mental health risks (Choukas-Bradley et al., 2022, Orben et al., 2024, Silk et al., 2024).

Taken together, these studies illustrate how research teams with intervention expertise in specific areas can empirically test actionable insights. Moreover, in this example, messaging that is sensitive to adolescents’ desire for admiration and respect, is also consistent with the heuristic of motivational salience for positive social value feedback. These examples demonstrate how a relatively nuanced affective construct (i.e., insights about sensitivity to feeling respected or valued) can translate into pragmatic progress in behavioral interventions.

Similar approaches can be utilized to design interventions to test features of a model focusing on a sensitive window for prosocial learning. Collaborative teams with expertise in interventions and implementation can help to identify ways to effectively increase opportunities for prosocial learning in specific contexts and to provide scaffolding/support to make it more likely for adolescents to have *successful* formative social learning experiences.

This type of approach can be informed by integrative learning models that focus on supporting students’ agency and scaffolded learning and discovery (Yeager et al., 2018, Yeager et al., 2014, Yeager et al., 2017). Attention to assessing *authentic* feelings of mattering and agency as active ingredients in interventions may be crucial. Focusing on affective processes may also provide important insights into how adolescents can explore and discover ways of contributing that feel personally and culturally meaningful and rewarding.

Consider, for example, the range of possible moment-to-moment affective experiences for a group of young adolescents who decide to volunteer in a shelter providing food and support for unhoused families. This could evoke some uncomfortable feelings, depending on the specific experiences as well as the background, attitudes, values, and motivations of the individual volunteers. As highlighted by one recent study of adolescent volunteerism, the contrast between extrinsic versus intrinsic motivations appears to be one particularly important dimension (Kwan & Wray-Lake, 2023). It is easy to imagine how an adolescent who is complying with a compulsory school requirement for volunteering, or who reports being motivated by the goal of ‘adding value to their CV’ might have different set of affective experiences than an adolescent who reports “helping people makes me happy”.

Transdisciplinary teams with the goal of designing interventions to increase opportunities for adolescents to have successful formative prosocial learning experiences could consider several different factors that could increase the likelihood of activating authentic feelings of mattering and caring. Such teams could benefit from the extensive real world experiences of groups who have trained and scaffolded adolescent volunteers (Hernantes et al., 2020, Wall, 2022). Programs that help prepare and support volunteers to develop helping skills and to perform supportive actions effectively, are more likely to result in authentic feelings of contribution and feelings of mattering. These successful (and rewarding) actions may also engender motivating feelings of caring and empathy (Pfister et al., 2024).

Another promising area of focus for designing interventions could build on insights about the onset of adolescence as a time of increased proclivities for *relational* learning. This may create opportunities to leverage these motivations through intervention designs with peers and mentors.

For example, pairing together near-peers in an intervention to promote exploration and discovery of prosocial learning. This could create opportunities for the older peer to gain feelings of admiration and mattering by helping the younger peer; who gains opportunities to learn and practice prosocial behaviors by emulating an older peer they admire (Qua et al., 2020).

Another example is an intervention that helped teachers learn an autonomy-supportive style of motivating middle and high school students that increased students’ exploration and engagement with prosocial behavior (Cheon et al., 2019). Given that girls often take on relatively more caregiving and household tasks compared to boys, encouraging young boys to participate in meaningful caregiving relationships could foster prosocial behavior from a young age. Indeed, given that adolescent boys face high levels of risk for antisocial behavior and often have limited opportunities to gain authentic feelings of mattering for prosocial actions, such efforts may help to prevent negative trajectories and promote holistic wellbeing in the ways we have described.

It is also important to recognize how the contemporary social learning environments of adolescents are characterized by nearly constant access to smart phones and social media (Pew Research Center, 2018). In this context, transdisciplinary teams, including expertise from computational modeling and human computer interactions, as well as developmental and learning sciences and social policy, may contribute unique perspectives to applying models, refining hypotheses, and applying these to large, scalable, testable interventions (Sultan et al., 2023).

While risks associated with social media are well documented, (Orben, 2020) digital environments also may provide unique opportunities for formative prosocial development through discovering and practicing prosocial ways of mattering. During the transition to adolescence, young people often gain or increase access to social media (Wait Until 8th, 2024). They often are beginning to expand ways of socially comparing themselves to others and question and explore their social value in new ways. Given that social media can provide ‘artificial’ short-term sensations of engagement, liking or re-posting prosocial content, leveraging social media in ways that offer more substantive opportunities for direct, engaged and meaningful prosocial action may be especially formative.

Technology based social interactions *could* provide expanded opportunities for prosocial learning. For example, learning to contribute to the lives of others in fundamentally new ways online, including asynchronously (Armstrong-Carter and Telzer, 2021, Nesi et al., 2018, Nesi et al., 2018, Odgers et al., 2009). The dynamic and rapidly changing nature of digital environments may offer unique opportunities for innovative approaches for scalable interventions—such as promoting collective prosocial actions for groups wanting to contribute to personally meaningful causes such as climate change mitigation or social justice (Armstrong‐Carter & Telzer, 2021).

This could include collaborative efforts with communities, practitioners, schools, and neighborhoods (and co-design efforts with youth) to devise ways to effectively support and scaffold opportunities for adolescents to engage meaningfully in collective action. Understanding and prioritizing the social and affective aspects of these experiences could increase the likelihood of formative prosocial learning. For example, adolescents who feel inspired by contributing to climate change mitigation through innovative approaches to changing social norms and behaviors, might entail day-to-day experiences that deepen individual feelings of caring about climate change, amplify feelings of motivation for continued actions, build peer-relationships with like-minded activists, and contribute to a social identity as a climate change activist. Testing features of these social learning models within intervention designs could not only advance scientific understanding but also improve the effectiveness of intervention approaches.

Another exciting frontier focuses on the interface of research integrating understanding of the developing learning systems *and* research aimed at intervention and policy. For example, advancing more integrative models of reinforcement learning (as described earlier) could be particularly important for understanding how social learning occurs in the context of social media and technology platforms—for example the amplification of uncertainty in ways that appear particularly relevant to adolescents, social learning, and relational learning (Ferguson et al., 2024).

Finally, one compelling framework for understanding the social and affective learning experiences of contemporary adolescents requires consideration of ongoing socioeconomic inequalities—particularly from a global perspective. Adolescent sensitivities to feeling valued, respected, and seeking opportunities to contribute in valued ways presents extreme challenges for youth growing up amidst poverty and socioeconomic hardship. This highlights the crucial importance of identifying policies that can leverage windows of opportunity to improve developmental trajectories during the pivotal transition to adolescence.

## 10 Coda

As described in the introduction, one of the goals of a more integrative understanding is to inform real-world efforts to improve developmental trajectories among the more than a billion adolescents growing up in complex and rapidly changing societies. Insights into opportunities for potentially pivotal changes in prosocial learning and mattering may be particularly relevant. The vast majority of these billion adolescents are growing up in low-to-middle income regions of the world—where they often face growing competition for *diminishing* resources. These contexts tend to kindle increasingly fierce competition for limited opportunities to earn money or gain status.

Yet, there are, potentially, *unlimited* opportunities for meaningful contribution. Including a diverse range of ways for adolescents to make a difference in some small or large way. Opportunities for helping others or contributing at a community or societal level are *not* diminishing in number.

Quite the opposite really. There is a growing abundance of needs and challenges in the world. There are multitudes—of individuals, communities, organizations, and societies—who could benefit greatly from (and gratefully appreciate) the innovative and inspired contributions of youth.

This perspective raises some compelling scientific and societal questions. *Why* is there such a chasm of disconnect between youth potential and youth engagement? Why do so many bright and talented adolescents—who *want* to contribute and to matter—struggle so often with feelings that they (and their actions) don’t really matter? How can scientific insights inform efforts to bridge this chasm? How can we design (and evaluate) interventions and policies to create greater opportunities for young adolescents to contribute in valued ways? In ways that allow them to discover authentic feelings of mattering. In ways that create deeply rewarding formative learning experiences that motivate further actions toward personally meaningful prosocial goals.

How might we leverage related scientific insights about windows of opportunity for *learning to care?* To more effectively encourage and support ways for adolescents learning how to care for and about each other. How might we engage youth to contribute to innovative approaches for strengthening social connectedness in our communities. Relevant to repairing what many have described as the fraying of the social fabric—what the U.S. Surgeon General has warned is the source of a public health crisis rooted in loneliness, isolation, and disconnection (Office of the Surgeon General, 2021).

These might seem like vastly complex challenges—far beyond the traditional scope of our science. And yet, why not, at least consider how we can contribute? Perhaps, by working collaboratively in transdisciplinary teams to provide new insights that can leverage windows of opportunity to improve developmental trajectories. In ways that can inform scalable positive impact. Perhaps, by discovering how to more effectively engage adolescents as valued partners in shaping a positive future—in ways that not only benefit their healthy social development, but also benefit society.

## CRediT authorship contribution statement

**Wouter van den Bos:** Writing – review & editing, Conceptualization. **Emma Armstrong-Carter:** Writing – review & editing, Writing – original draft, Conceptualization. **Ronald E Dahl:** Writing – review & editing, Writing – original draft, Project administration, Funding acquisition, Conceptualization.

## Declaration of Competing Interest

The authors declare that they have no known competing financial interests or personal relationships that could have appeared to influence the work reported in this paper.

## References

[bib1] Achterberg M., Van Duijvenvoorde A.C.K., Van Der Meulen M., Euser S., Bakermans-Kranenburg M.J., Crone E.A. (2017). The neural and behavioral correlates of social evaluation in childhood. Dev. Cogn. Neurosci..

[bib2] Allen J.P., Costello M.A., Hellwig A.F., Pettit C., Stern J.A., Uchino B.N. (2023). Adolescent caregiving success as a predictor of social functioning from ages 13 to 33. Child Dev..

[bib3] Anderson G.M., Hill J.W., Kaiser U.B. (2024). Metabolic control of puberty: 60 years in the footsteps of Kennedy and Mitra’s seminal work. Nat. Rev. Endocrinol..

[bib4] Armstrong-Carter E., Johnson C., Belkowitz J., Siskowski C., Olson E. (2021). The United States should recognize and support caregiving youth. Soc. Policy Rep..

[bib5] Armstrong-Carter E., Olson E., Telzer E. (2019). A unifying approach for investigating and understanding youth’s help and care for the family. Child Dev. Perspect..

[bib6] Armstrong-Carter E., Telzer E.H. (2021). Advancing measurement and research on youths’ prosocial behavior in the digital age. Child Dev. Perspect..

[bib7] Bach D.R., Dayan P. (2017). Algorithms for survival: a comparative perspective on emotions. Nat. Rev. Neurosci..

[bib8] Barrett K.C., Morgan G.A. (2018).

[bib9] Berridge K.C. (1996). Food reward: brain substrates of wanting and liking. Neurosci. Biobehav Rev..

[bib10] Berridge K.C. (2019). Affective valence in the brain: modules or modes?. Nat. Rev. Neurosci..

[bib11] Biele G., Rieskamp J., Gonzalez R. (2009). Computational models for the combination of advice and individual learning. Cogn. Sci..

[bib12] Bigelow A.E., Rochat P. (2006). Two-month-old infants’ sensitivity to social contingency in mother–infant and stranger–infant interaction. Infancy.

[bib13] Blake P.R., McAuliffe K., Corbit J. (2015). The ontogeny of fairness in seven societies. Nature.

[bib14] Blakemore S.J. (2008). The social brain in adolescence. Nat. Rev. Neurosci..

[bib15] Blakemore S., Burnett S., Dahl R.E. (2010). The role of puberty in the developing adolescent brain. Hum. Brain Mapp..

[bib16] Blakemore S.J., Mills K.L. (2014). Is adolescence a sensitive period for sociocultural processing?. Annu Rev. Psychol..

[bib17] Brownell C.A., Svetlova M., Anderson R., Nichols S.R., Drummond J. (2013). Socialization of early rosocial behavior: parents’ talk about emotions is associated with sharing and helping in toddlers. Infancy.

[bib18] Bryan C.J., Yeager D.S., Hinojosa C.P. (2019). A values-alignment intervention protects adolescents from the effects of food marketing. Nat. Hum. Behav..

[bib19] Capella J., Jorgensen N.A., Kwon S.J. (2023). Adolescents’ neural sensitivity to high and low popularity: longitudinal links to risk-taking and prosocial behavior. Dev. Cogn. Neurosci..

[bib20] Cardoos S.L., Ballonoff Suleiman A., Johnson M., Van Den Bos W., Hinshaw S.P., Dahl R.E. (2017). Social status strategy in early adolescent girls: testosterone and value-based decision making. Psychoneuroendocrinology.

[bib21] Caregiving Youth Research Collaborative. Report on caregiving youth in the US: Progress and opportunity. Published 2023. Accessed December 28, 2023. https://aacy.org/wp-content/uploads/2023/12/CY-White-Paper_Final-.pdf.

[bib22] Carlo G. (2022). The development and correlates of prosocial moral behaviors. Handb. Moral Dev..

[bib23] Carlo G., Padilla-Walker L. (2020). Adolescents’ prosocial behaviors through a multidimensional and multicultural lens. Child Dev. Perspect..

[bib24] Chase-Lansdale P.L., Wakschlag L.S., Brooks-Gunn J. (1995). A psychological perspective on the development of caring in children and youth: the role of the family. J. Adolesc..

[bib25] Cheon S.H., Reeve J., Ntoumanis N. (2019). An intervention to help teachers establish a prosocial peer climate in physical education. Learn Instr..

[bib26] Choi B.C.K., P A.W. (2008). Multidisciplinarity, interdisciplinarity, and transdisciplinarity in health research, services, education and policy: 3. Discipline, inter-discipline distance, and selection of discipline. Clin. Invest Med.

[bib27] Choi B.C.K., Pak A.W.P. (2006). Multidisciplinarity, interdisciplinarity and transdisciplinarity in health research, services, education and policy: 1. definitions, objectives, and evidence of effectiveness. Clin. Invest. Med Med Clin. Exp..

[bib28] Choi B.C.K., Pak A.W.P. (2007). Multidisciplinarity, interdisciplinarity, and transdisciplinarity in health research, services, education and policy: 2. Promotors, barriers, and strategies of enhancement. Clin. Invest Med.

[bib29] Choukas-Bradley S., Roberts S.R., Maheux A.J., Nesi J. (2022). The perfect storm: a developmental–sociocultural framework for the role of social media in adolescent girls’ body image concerns and mental health. Clin. Child Fam. Psychol. Rev..

[bib30] Ciesielski T.H., Aldrich M.C., Marsit C.J., Hiatt R.A., Williams S.M. (2017). Transdisciplinary approaches enhance the production of translational knowledge. Transl. Res.

[bib31] Ciranka S., van den Bos W. (2019). Social influence in adolescent decision-making: a formal framework. Front Psychol..

[bib32] Crone E.A., Achterberg M., Dobbelaar S. (2020). Neural and behavioral signatures of social evaluation and adaptation in childhood and adolescence: the leiden consortium on individual development (L-CID). Dev. Cogn. Neurosci..

[bib33] Crone E.A., Bol T., Braams B.R. (June 2024). Dev Cogn Neurosci.

[bib34] Crone E.A., Dahl R.E. (2012). Understanding adolescence as a period of social–affective engagement and goal flexibility. Nat. Rev. Neurosci..

[bib35] Crone E.A., Fuligni A.J. (2020). Self and others in adolescence. Annu Rev. Psychol..

[bib36] Curran T., Hill A.P. (2022). Young people’s perceptions of their parents’ expectations and criticism are increasing over time: Implications for perfectionism. Psychol. Bull..

[bib37] Dahl R.E., Allen N.B., Wilbrecht L., Suleiman A.B. (2018). Importance of investing in adolescence from a developmental science perspective. Nature.

[bib38] Dai J., Jorgensen N.A., Duell N. (2023). Neural tracking of social hierarchies in adolescents’ real-world social networks. Soc. Cogn. Affect Neurosci..

[bib39] Dalgleish T., Walsh N.D., Mobbs D. (2017). Social pain and social gain in the adolescent brain: a common neural circuitry underlying both positive and negative social evaluation. Sci. Rep..

[bib40] Davis M.M., Modi H.H., Skymba H.V. (2023). Thumbs up or thumbs down: neural processing of social feedback and links to social motivation in adolescent girls. Soc. Cogn. Affect Neurosci..

[bib41] Demir A., Hero M., Juul A., Main K.M. (2023). Sex-independent timing of the onset of central puberty revealed by nocturnal luteinizing hormone concentrations. Clin. Endocrinol. (Oxf. ).

[bib42] Dobbelaar S., Achterberg M., Van Drunen L., Van Duijvenvoorde A.C.K., Van IJzendoorn M.H., Crone E.A. (2023). Development of social feedback processing and responses in childhood: an fMRI test-replication design in two age cohorts. Soc. Cogn. Affect Neurosci..

[bib43] Dobbelaar S., Achterberg M., Van Duijvenvoorde A.C.K., Van IJzendoorn M.H., Crone E.A. (2023). Developmental patterns and individual differences in responding to social feedback: a longitudinal fMRI study from childhood to adolescence. Dev. Cogn. Neurosci..

[bib44] Durlach P.J., Elliman N.A., Rogers P.J. (2002). Drinking while thirsty can lead to conditioned increases in consumption. Appetite.

[bib45] East P.L. (2010). Children’s provision of family caregiving: benefit or burden?. Child Dev. Perspect..

[bib46] Eisenberg N., Spinrad T.L., Knafo-Noam A. (2015). Prosocial Dev.

[bib47] Eisenberg N., VanSchyndel S.K., Spinrad T.L. (2016). Prosocial motivation: Inferences from an opaque body of work. Child Dev..

[bib48] Eldar E., Rutledge R.B., Dolan R.J., Niv Y. (2016). Mood as representation of momentum. Trends Cogn. Sci..

[bib49] Elliott G.C., Colangelo M.F., Gelles R.J. (2005). Mattering and suicide ideation: establishing and elaborating a relationship. Soc. Psychol. Q.

[bib50] Emanuel A., Eldar E. (2023). Emotions as computations. Neurosci. Biobehav Rev..

[bib51] Ferguson A.M., Turner G., Orben A. (2024). Trends Cogn Sci.

[bib52] Flett (2018).

[bib53] Flett L., Flett A., Wekerle C. (2016). A conceptual analysis of interpersonal resilience as a key resilience domain: understanding the ability to overcome child sexual abuse and other adverse interpersonal contexts. Int J. Child Adolesc. Resil..

[bib54] Flett G., Nepon T., Goldberg J.O., Rose A.L., Atkey S.K., Zaki-Azat J. (2022). The anti-mattering scale: development, psychometric properties and associations with well-being and distress measures in adolescents and emerging adults. J. Psychoeduc. Assess..

[bib55] Fong H., Zheng J., Kurrasch D. (2023). The structural and functional complexity of the integrative hypothalamus. Science.

[bib56] Foulkes L., Leung J.T., Fuhrmann D., Knoll L.J., Blakemore S.J. (2018). Age differences in the prosocial influence effect. Dev. Sci..

[bib57] Fuligni A.J. (2019). The need to contribute during adolescence. Perspect. Psychol. Sci..

[bib58] Gaby S. (2017). The civic engagement Gap(s): youth participation and inequality from 1976 to 2009. Youth Soc..

[bib59] Galla B.M., Choukas-Bradley S., Fiore H.M., Esposito M.V. (2021). Values-alignment messaging boosts adolescents’ motivation to control social media use. Child Dev..

[bib60] Grueneisen S., Warneken F. (2022). The development of prosocial behavior—from sympathy to strategy. Curr. Opin. Psychol..

[bib61] Gunther Moor B., Van Leijenhorst L., Rombouts S.A.R.B., Crone E.A., Van Der Molen M.W. (2010). Do you like me? Neural correlates of social evaluation and developmental trajectories. Soc. Neurosci..

[bib62] Hall W.G., Arnold H.M., Myers K.P. (2000). The acquisition of an appetite. Psychol. Sci..

[bib63] Hartl A.C., Laursen B., Cantin S., Vitaro F. (2020). A test of the bistrategic control hypothesis of adolescent popularity. Child Dev..

[bib64] Hawley P.H. (2003). Prosocial and coercive configurations of resource control in early adolescence: a case for the well-adapted machiavellian. Merrill-Palmer Q.

[bib65] Hawley P.H. (2014). The duality of human nature: coercion and prosociality in youths’ hierarchy ascension and social success. Curr. Dir. Psychol. Sci..

[bib66] Hawley P.H., Little T.D., Card N.A. (2007). The allure of a mean friend: relationship quality and processes of aggressive adolescents with prosocial skills. Int J. Behav. Dev..

[bib67] Hensums M., Brummelman E., Larsen H., Van Den Bos W., Overbeek G. (2023). Social goals and gains of adolescent bullying and aggression: a meta-analysis. Dev. Rev..

[bib68] Hepach R., Engelmann J.M., Herrmann E., Gerdemann S.C., Tomasello M. (2023). Evidence for a developmental shift in the motivation underlying helping in early childhood. Dev. Sci..

[bib69] Hernantes N., Pumar-Méndez M.J., López-Dicastillo O., Iriarte A., Mujika A. (2020). Volunteerism as adolescent health promotion asset: a scoping review. Health Promot Int.

[bib70] Higgins E.T. (2016). Shared-reality development in childhood. Perspect. Psychol. Sci..

[bib71] Hooper L.M., L’Abate L., Sweeney L.G., Gianesini G., Jankowski P.J. (2014). Models of Psychopathology.

[bib72] Hrdy S.B. Mothers and Others: The Evolutionary Origins of Mutual Understanding. 1st Harvard University Press paperback ed. Belknap Press of Harvard University Press; 2011.

[bib73] Jabarin R., Dagash W., Netser S. (2023). Modulation of social investigation by anterior hypothalamic nucleus rhythmic neural activity. iScience.

[bib74] James A.S., Gehlert S., Bowen D.J., Colditz G.A. (2015). A framework for training transdisciplinary scholars in cancer prevention and control. J. Cancer Educ..

[bib75] Jara-Ettinger J. (2019). Theory of mind as inverse reinforcement learning. Curr. Opin. Behav. Sci..

[bib76] Juechems K., Summerfield C. (2019). Where does value come from?. Trends Cogn. Sci..

[bib77] Jung A.K., Heppner M.J. (2017). Development and validation of a work mattering scale (WMS). J. Career Assess..

[bib78] Kendal R.L., Boogert N.J., Rendell L., Laland K.N., Webster M., Jones P.L. (2018). Social learning strategies: bridge-building between fields. Trends Cogn. Sci..

[bib79] Keramati M., Gutkin B. (2014). Homeostatic reinforcement learning for integrating reward collection and physiological stability. eLife.

[bib80] Kwan J.Y., Wray-Lake L. (2023). Asia Pac J Educ.

[bib81] LaFontana K.M., Cillessen A.H.N. (2010). Developmental changes in the priority of perceived status in childhood and adolescence. Soc. Dev..

[bib82] Lam-Cassettari C., Peter V., Antoniou M. (2021). Babies detect when the timing is right: evidence from event-related potentials to a contingent mother-infant conversation. Dev. Cogn. Neurosci..

[bib83] Lockwood P., Van Den Bos W., Dreher J.C. (2024).

[bib84] Lucien J.N., Ortega M.T., Shaw N.D. (2021). Sleep and puberty. Curr. Opin. Endocr. Metab. Res.

[bib85] Luthar S.S., Kumar N.L., Zillmer N. (2020). High-achieving schools connote risks for adolescents: problems documented, processes implicated, and directions for interventions. Am. Psychol..

[bib86] Markus H.R., Kitayama S. (1991). Culture and the self: Implications for cognition, emotion, and motivation. Psychol. Rev..

[bib87] Maroso M., Stern P. (2023). Small and mighty: the hypothalamus. Science.

[bib88] Masek L.R., McMillan B.T.M., Paterson S.J., Tamis-LeMonda C.S., Golinkoff R.M., Hirsh-Pasek K. (2021). Where language meets attention: how contingent interactions promote learning. Dev. Rev..

[bib89] Masten C.L., Eisenberger N.I., Pfeifer J.H., Dapretto M. (2013). Neural responses to witnessing peer rejection after being socially excluded: fMRI as a window into adolescents’ emotional processing. Dev. Sci..

[bib90] Matera C., Bosco N., Meringolo P. (2020). Perceived mattering to family and friends, self-esteem, and well-being. Psychol. Health Med.

[bib91] McMakin D.L., Alfano C.A. (2015). Sleep and anxiety in late childhood and early adolescence. Curr. Opin. Psychiatry.

[bib92] Menon V., Uddin L.Q. (2010). Saliency, switching, attention and control: a network model of insula function. Brain Struct. Funct..

[bib93] Minges K.E., Redeker N.S. (2016). Delayed school start times and adolescent sleep: a systematic review of the experimental evidence. Sleep. Med Rev..

[bib94] Molleman L., Ciranka S., Van Den Bos W. (2022). Social influence in adolescence as a double-edged sword. Proc. R. Soc. B Biol. Sci..

[bib95] Molnar-Szakacs I., Uddin L.Q. (2022). Anterior insula as a gatekeeper of executive control. Neurosci. Biobehav Rev..

[bib96] Morgenthaler T.I., Hashmi S., Croft J.B., Dort L., Heald J.L., Mullington J. (2016). High school start times and the impact on high school students: what we know, and what we hope to learn. J. Clin. Sleep. Med.

[bib97] Naulé L., Maione L., Kaiser U.B. (2021). Puberty, a sensitive window of hypothalamic development and plasticity. Endocrinology.

[bib98] Nelson E.E., Jarcho J.M., Guyer A.E. (2016). Social re-orientation and brain development: an expanded and updated view. Dev. Cogn. Neurosci..

[bib99] Nelson E.E., Leibenluft E., McCLURE E.B., Pine D.S. (2005). The social re-orientation of adolescence: a neuroscience perspective on the process and its relation to psychopathology. Psychol. Med.

[bib100] Nesi J. (2024). Techno Sapiens.

[bib101] Nesi J., Choukas-Bradley S., Prinstein M.J. (2018). Transformation of adolescent peer relations in the social media context: Part 2—application to peer group processes and future directions for research. Clin. Child Fam. Psychol. Rev..

[bib102] Nesi J., Choukas-Bradley S., Prinstein M.J. (2018). Transformation of adolescent peer relations in the social media context: Part 1—a theoretical framework and application to dyadic peer relationships. Clin. Child Fam. Psychol. Rev..

[bib103] Nowell C., Pfeifer J.H., Enticott P., Silk T., Vijayakumar N. (2023). Value of self-disclosure to parents and peers during adolescence. J. Res Adolesc..

[bib104] Nussenbaum K., Hartley C.A. (2019). Reinforcement learning across development: what insights can we draw from a decade of research?. Dev. Cogn. Neurosci..

[bib105] O’Byrne N.A., Yuen F., Butt W.Z., Liu P.Y. (2021). Sleep and circadian regulation of cortisol: a short review. Curr. Opin. Endocr. Metab. Res..

[bib106] Odgers C.L., Moffitt T.E., Tach L.M. (2009). The protective effects of neighborhood collective efficacy on British children growing up in deprivation: a developmental analysis. Dev. Psychol..

[bib107] Office of the Surgeon General (OSG). Protecting Youth Mental Health: The U.S. Surgeon General’s Advisory. US Department of Health and Human Services; 2021. Accessed March 5, 2024. http://www.ncbi.nlm.nih.gov/books/NBK575984/. 34982518

[bib108] Olsson A., Knapska E., Lindström B. (2020). The neural and computational systems of social learning. Nat. Rev. Neurosci..

[bib109] Orben A. (2020). Teenagers, screens and social media: a narrative review of reviews and key studies. Soc. Psychiatry Psychiatr. Epidemiol..

[bib110] Orben A., Meier A., Dalgleish T., Blakemore S.J. (2024). Nat Rev Psychol.

[bib111] Paz Y., Davidov M., Orlitsky T., Hayut M., Roth-Hanania R., Zahn-Waxler C. (2023). Prosocial behavior in toddlerhood and early childhood: consistency across subtypes and over time. Front Psychol..

[bib112] Pelletier-Baldelli A., Sheridan M.A., Rudolph M.D. (2024). Brain network connectivity during peer evaluation in adolescent females: associations with age, pubertal hormones, timing, and status. Dev. Cogn. Neurosci..

[bib113] Perino M.T., Miernicki M.E., Telzer E.H. (2016). Letting the good times roll: adolescence as a period of reduced inhibition to appetitive social cues. Soc. Cogn. Affect Neurosci..

[bib114] Petzold A., Van Den Munkhof H.E., Figge-Schlensok R., Korotkova T. (2023). Complementary lateral hypothalamic populations resist hunger pressure to balance nutritional and social needs. Cell Metab..

[bib115] Pew Research Center. Teens, social media & technology 2018. Pew Research Center: Internet, Science & Tech. Published 2018. Accessed September 11, 2020. https://www.pewresearch.org/internet/2018/05/31/teens-social-media-technology-2018/.

[bib116] Pfeifer J.H., Berkman E.T. (2018). The development of self and identity in adolescence: neural evidence and implications for a value-based choice perspective on motivated behavior. Child Dev. Perspect..

[bib117] Pfister T.A., Rimm-Kaufman S.E., Deutsch N.L., Sandilos L.E. (2024). “The most important part of empathy is…being able to help”: empathy definitions and teaching practices in middle school. Appl. Dev. Sci..

[bib118] Qua K., Pinkard O., Kundracik E.C., Ramirez-Bergeron D., Berger N.A. (2020). Near peer mentors to address socio-emotional issues among underrepresented minority high school students in research intensive STEM programs: perceptions of students and mentors. J. STEM Outreach.

[bib119] Reece A., Yaden D., Kellerman G. (2021). Mattering is an indicator of organizational health and employee success. J. Posit. Psychol..

[bib120] Rochat P.R. (2001). Social contingency detection and infant development. Bull. Menn. Clin..

[bib121] Rodriguez Buritica J.M., Heekeren H.R., Van Den Bos W. (2019). The computational basis of following advice in adolescents. J. Exp. Child Psychol..

[bib122] Rogoff B. (2014). Learning by observing and pitching in to family and community endeavors: an orientation. Hum. Dev..

[bib123] Rosenberg M. (1965). Rosenberg self-esteem scale. Publ. Online.

[bib124] Rosenberg M., McCullough B. (1981). Mattering: inferred significance and mental health among adolescents. Res Community Ment. Health.

[bib125] Sadeh A., Dahl R.E., Shahar G., Rosenblat-Stein S. (2009). Sleep and the transition to adolescence: a longitudinal study. Sleep.

[bib126] Schlossberg N.K. (1989). Marginality and mattering: key issues in building community. N. Dir. Stud. Serv..

[bib127] Seeley W.W. (2019). The salience network: a neural system for perceiving and responding to homeostatic demands. J. Neurosci..

[bib128] Seeley W.W., Menon V., Schatzberg A.F. (2007). Dissociable intrinsic connectivity networks for salience processing and executive control. J. Neurosci..

[bib129] Sell K., Hommes F., Fischer F., Arnold L. (2022). Multi-, inter-, and transdisciplinarity within the public health workforce: a scoping review to assess definitions and applications of concepts. Int J. Environ. Res Public Health.

[bib130] Silk J.B., House B.R. (2011). Evolutionary foundations of human prosocial sentiments. Proc. Natl. Acad. Sci..

[bib131] Silk J.S., Sequeira S.L., James K.M. (2024). The Role of Neural Sensitivity to Social Evaluation in Understanding “for Whom” Social Media Use May Impact Emotional Health During Adolescence. Affec Sci.

[bib132] Silk J.S., Siegle G.J., Lee K.H., Nelson E.E., Stroud L.R., Dahl R.E. (2014). Increased neural response to peer rejection associated with adolescent depression and pubertal development. Soc. Cogn. Affect Neurosci..

[bib133] Silk J.S., Stroud L.R., Siegle G.J., Dahl R.E., Lee K.H., Nelson E.E. (2012). Peer acceptance and rejection through the eyes of youth: pupillary, eyetracking and ecological data from the Chatroom Interact task. Soc. Cogn. Affect Neurosci..

[bib134] Siskowski C. (2006). Young caregivers: effect of family health situations on school performance. J. Sch. Nurs..

[bib135] Somerville L.H. (2013). The teenage brain: sensitivity to social evaluation. Curr. Dir. Psychol. Sci..

[bib136] Song Y., Broekhuizen M., Dubas J.S. (2022). A three-wave study on the development of prosocial behaviours across toddlerhood: the role of socialization. Infant Child Dev..

[bib137] Suleiman A.B., Galván A., Harden K.P., Dahl R.E. (2017). Becoming a sexual being: the ‘elephant in the room’ of adolescent brain development. Dev. Cogn. Neurosci..

[bib138] Sultan M., Scholz C., Van Den Bos W. (2023). Leaving traces behind: using social media digital trace data to study adolescent wellbeing. Comput. Hum. Behav. Rep..

[bib139] Telzer E.H., Jorgensen N.A., Prinstein M.J., Lindquist K.A. (2021). Neurobiological sensitivity to social rewards and punishments moderates link between peer norms and adolescent risk taking. Child Dev..

[bib140] Tomasello M. (2018). The normative turn in early moral development. Hum. Dev..

[bib141] Tomasello M. (2019).

[bib142] Tomasello M., Carpenter M. (2007). Shared intentionality. Dev. Sci..

[bib143] Tomasello M., Vaish A. (2013). Origins of human cooperation and morality. Annu Rev. Psychol..

[bib144] Van De Groep I.H., Bos M.G.N., Jansen L.M.C., Achterberg M., Popma A., Crone E.A. (2021). Overlapping and distinct neural correlates of self-evaluations and self-regulation from the perspective of self and others. Neuropsychologia.

[bib145] Van Der Meulen M., Dobbelaar S., Van Drunen L. (2023). Transitioning from childhood into adolescence: a comprehensive longitudinal behavioral and neuroimaging study on prosocial behavior and social inclusion. NeuroImage.

[bib146] Vélez C.E., Braver S.L., Cookston J.T., Fabricius W.V., Parke R.D. (2020). Does mattering to parents matter to adolescent mental health?: a psychometric analysis. Fam. Relat..

[bib147] Vernetti A., Senju A., Charman T., Johnson M.H., Gliga T. (2018). Simulating interaction: using gaze-contingent eye-tracking to measure the reward value of social signals in toddlers with and without autism. Dev. Cogn. Neurosci..

[bib148] Vollberg M.C., Sander D. (2024). Curr Dir Psychol Sci.

[bib149] Wait Until 8th. Let Kids Be Kids a Little Longer.; 2024. https://www.waituntil8th.org/.

[bib150] Wall J. What Can You Learn From Volunteering At A Food Bank? | Be a Volunteer Today. Second Harvest of the Greater Valley. Published April 22, 2022. Accessed June 19, 2024. https://localfoodbank.org/what-do-you-learn-from-volunteering-at-a-food-bank/.

[bib151] Warneken F. (2016). Insights into the biological foundation of human altruistic sentiments. Curr. Opin. Psychol..

[bib152] Warneken F., Tomasello M. (2009). The roots of human altruism. Br. J. Psychol..

[bib153] Watson J., Ramey C. (1972). Reactions to response-contingent stimulation in early infancy. Merrill-Palmer Q Behav. Dev..

[bib154] Wilbrecht L., Davidow J.Y. (2024). Nat Rev Neurosci.

[bib155] Wolf W., Tomasello M. (2023). Perspect Psychol Sci.

[bib156] Yeager D.S., Dahl R.E., Dweck C.S. (2018). Why interventions to influence adolescent behavior often fail but could succeed. Perspect. Psychol. Sci..

[bib157] Yeager D.S., Henderson M.D., Paunesku D. (2014). Boring but important: a self-transcendent purpose for learning fosters academic self-regulation. J. Pers. Soc. Psychol..

[bib158] Yeager D., Lee H., Dahl R., Elliot A.J., Dweck C.S., Yeager D.S. (2017). Handbook of Competence and Motivation: Theory and Application.

